# Optimal Design of Language Teaching Strategy System Based on Smart Video Mode

**DOI:** 10.1155/2022/7501765

**Published:** 2022-06-28

**Authors:** Jia Liu, Jianling Guo

**Affiliations:** Department of Foundational Disciplines, Shijiazhuang People's Medical College, Shijiazhuang, Hebei, China

## Abstract

The accuracy of video and goal enables students to learn and strengthen their ability constantly. Strengthening our country's study initiative degree can promote student study more effectively. As a new teaching method, students can not only obtain the basic knowledge, learning priorities, and difficulties needed for learning through video, but also understand the content of the text, the content of the article, and even cultivate students' interest in many related languages, such as writing, text, sound, image, color, and video, which can be displayed, clarified, and displayed intuitively, creating a free and relaxed learning environment, an interesting background teaching process, encouraging students to experience emotion, including physical experience, and being open and open. Establishing a complete and comprehensive ideological channel to further improve students' acceptance of information is helpful for students' analysis and training, understanding, and evaluation. Therefore, this paper first identifies video and excavates the intrinsic value of video application. This can provide technical and methodological support for the design of video teaching system.

## 1. Introduction

The accuracy of video and goals enables students to continuously learn and strengthen their abilities [1]. The concept of video learning was first proposed in the mid-1990s, and Carnegie Mellon University took the lead in introducing the concept of video learning into a research project, which was reorganized, understood and promoted, and approved [2]. At the same time, more and more educators begin to pay attention to video learning and vigorously promote the application of the concept of video learning to practical education and reform traditional teaching methods, which leads to modern video learning models, such as distance learning and university education [3]. Because video learning is very useful and convenient, many scholars and educators have taken a very positive attitude towards video learning. At the same time, our country has kept pace with the times and has begun to vigorously promote the use of video learning in the national education model [4]. The Ministry of Education has taken educational initiatives, such as the theory and practice of video teaching and video teaching. In addition, the state also attaches importance to video teaching in colleges and universities. Many of the best educational and training enterprises in China, such as the new Oriental educational institutions and elite online education, have developed their own video learning platforms to enhance the competitiveness of educational and training institutions in the new information age [5]. Reform education, such as Chinese teaching, is usually carried out in the form of text materials, with abstract and complex characteristics; the application of traditional teaching methods can easily lead to the low interest, motivation, and interest. For Chinese learning, as a new form of teaching, students can not only acquire the basic knowledge needed for learning through videos, learning priorities, and challenges, but also understand the content of the text, the content of the article, including stimulating people's interest in writing, writing abstracts, through words, sounds, images, colors, and videos, clarify and vividly display the teaching content, create a free and relaxed learning environment, a learning process background, vision, and fun, encourage students to carry out emotional experiments, open up comprehensive and comprehensive channels of thought, and further improve students' acceptance of information. It is helpful for students to carry out analysis training and improve students' understanding and evaluation of teaching [6]. In this paper, starting from the visual recognition of video, the intrinsic value of video use is excavated to provide technical and methodological support for the design of video education system.

## 2. Related Works

About video teaching, the literature suggests that the University of Nottingham (UK) has done an excellent job in information exchange, and user teachers can access the My Art Space platform. And their understanding of the subject or their views on difficult issues are recorded to the My Art Space platform, so that students in need can access it. Meanwhile, students, as the main body of the My Art Space platform, can also bring their learning experience to the platform, and students can communicate on the My Art Space platform on specific subjects. The literature suggests that Harvard University (USA) attaches great importance to the information obtained from students about school education programs. Therefore, the Department of Educational Science at Harvard University specializes in studying the experience and views of each student on the professional courses offered and proposing Hudl projects. Through systematic analysis data on the 8.344 million, more course-compliant staff training programs collected information on the learning of different professional students in different school curricula. The literature of South Dakota University in the United States has invested in the design of a student self-study service platform to facilitate the preservation of learning materials and the search for learning resources, which is a good example of the application of video learning technology abroad. According to the literature, the typical practice of video teaching technology university is adopted in vocational training. In practice, medical school students often face particularly difficult situations. In order to provide relevant expertise or to respond appropriately to these difficult situations, the University of Oslo, Norway, has designed a manual called Know mobile to effectively address the learning and work problems of medical school students. Documentation presents the current situation in Australia, where the Development Group has expanded the concept of video learning, not just to train specialized technicians through online video learning platforms, but also creatively incorporated the concept of evaluation and evaluation into video learning as a means of monitoring the effectiveness of students' learning practices and outcomes. Document Birmingham University (UK) designed a video learning project called Handler to solve some practical problems, such as the concept of lifelong learning, which is deeply rooted in today's society, but students in different age groups often have different expectations for lifelong learning. The above examples of video learning applications show that institutions of higher learning, teaching research, and teaching institutions are the pillars of applying and promoting video learning technology, which is also proved by the application of video learning technology. It helps solve some defects in the traditional education model and meet the learning needs of the whole society.

For the design of video teaching strategy system, literature video is regarded as an integral part of “video teaching,” which is the alias of video teaching syllabus, focusing on classroom teaching video, and other supplementary resources, such as teaching design, textbooks, exercises, and tests, and teaching reflection [7]. Create a theme-centric, semistructured application environment for modular resources. Based on individualized learning content, teaching system element theory, and learning condition theory, a model of influencing factors determined in learning based on relevant national and international research results is developed [8]. The international model shows that students, environment, resources, and teachers are four factors that affect the effect of individualized learning. The explanation of the learning resource factor states that “designers or teachers must be able to integrate and select learning resources in advance according to the needs and interests of students,” so the presentation of the organization and resources should be in line with the learning styles and learning expectations of the organizations of the United Nations system. It ensures that students have the opportunity to acquire knowledge through online learning resources and promote the development of their learning ability. Literature provides learning choices for students in the network by studying and analyzing the behavior of students accessing platform resources and making suggestions on appropriate learning resources [9]. Video is defined as “a view resource consisting of a complete unit or knowledge point of multiple knowledge atoms, completed within 2 to 20 minutes” [10]. In order to study how the production of teaching video affects the teaching of program knowledge, four introductory videos are designed, namely, “graphic sketch,” “homework recording,” “graphic illustration + teacher,” and “homework recording and recording teacher,” which are summarized into a broadcast classroom mode. Some people point out that students' preference for teaching videos is inconsistent and needs to take into account students' cognitive habits [11, 12].

According to previous studies, it can be seen that the individuation of students' cognitive style in the process of learning is reflected in their understanding of information and the process of compiling information [13]. Although the design of curriculum resources takes into account the different preferences of students in the process of learning, the individuation of students' cognitive style in the process of learning is reflected in their understanding of information [14]. As for the problem of accepting resources in self-study on the Internet, there is no special design research on the satellite resources that accord with the individualized learning mode, and the video resources for personalized learning are less.

## 3. Video Scene Recognition Based on Artificial Intelligence

### 3.1. Multimodal Complementary Video Scene Retrieval Method

In the past few years, video has been widely spread in social networks as a new way of communication. Unlike traditional videos, video adds more social attributes, such as comments and topics. The text information contained in these social attributes is very useful for analysis and understanding. The characteristics of multimedia analysis and understanding show that semantic information may be better understood in some multimedia. In some cases, such as in a single mode, and in the tasks of social media (such as referral systems), social characteristics are often common feature information with other data. Considering the semantic richness of text information, text information can be described as a multimodule video scene feature, plus audio-visual information contained in the video itself.

However, space learning faces some challenges and problems when applied to video in real situations. The correlation between visual, sound, and text models of video is weak, but there is complementarity between different parts.  Table 1 provides the interrelationship between two modes: visual mode and the interrelationship between sound and text in size text between sound and text. Tambin analyzes the interrelationship between these three units and case categories, as given in Table 2; it can be seen from the table that the correlation between visual mode and category is greater. It can be seen from Table 3 that both unsupervised and supervised learning methods cannot fully reflect semantic information. These problems are called low correlation between different models. The video models with different relationships are weak and complementary. The integrated features of multimodal transport can be represented using supplementary information of multimodal transport as given in Tables 1–3.

#### 3.1.1. Method Description

Given the training set sample set *D*, each sample contains three modes of vision, sound, and text. During training, the dataset is divided into *N* batch inputs. The amount of data per batch is set to *b*_*n*_, the final loss function representation as shown in

(1) $$\begin{array}{c} E_{n}\left(W,B\right)=-\frac{1}{\text{bn}}\sum_{i=1}^{bn}yi\;\ln\;{\hat{y}}_{i}+\left(1-y_{i}\right)\ln\left(1-{\hat{y}}_{i}\right). \end{array}$$

This method uses the minimum batch gradient descent algorithm to optimize the network weight *W* and bias term *B*. Parameter updates are shown in formulas (3) and (4), respectively.

(2) $$\begin{array}{c} W=W-\eta_{n1}\frac{\partial E_{n}}{\partial W}, \end{array}$$

(3) $$\begin{array}{c} B=B-\eta_{n2}\frac{\partial E_{n}}{\partial B}. \end{array}$$

The loss of each batch is continuously adjusted by the backpropagation algorithm until the algorithm converges. Given the test set sample, it is input into the trained network structure, and the prediction results are obtained.

The objective function of learning is shown in

(4) $$\begin{array}{c} \underset{W,B}{\text{min}}E_{\text{h}}=|{\left\|Y-W^{T}\mathrm{sgn}\left(B\right)\right\|}^{2}+|{\left\|\text{S}-\frac{1}{t}\mathrm{sgn}{\left(B\right)}^{T}\mathrm{sgn}\left(B\right)\right\|}^{2}+\lambda {\left\|W\right\|}^{2}\\ s.t.B=A^{T}\hat{X}\\ \text{sgn}\left(B\right)\mathrm{sgn}{\left(B\right)}^{T}=I. \end{array}$$

Construction of the similarity matrix *S* is as follows:

(5) $$\begin{array}{c} s_{ij}=\left\{\begin{array}{cc} 1 & {\text{ify}}_{i}{\text{issimilartoy}}_{i}\\ -1 & \mathrm{else}\text{.} \end{array}\right\} \end{array}$$

For ease of solution, this section relaxes it as

(6) $$\begin{array}{c} \underset{W,B}{\min}E_{h}=||Y-W^{T}B|{|}^{2}+||S-\frac{1}{t}B^{T}B|{|}^{2}+\lambda ||W|{|}^{2}\\ \begin{array}{c} s.t. \end{array}BB^{T}=I. \end{array}$$

Since the closed solution of variable *B* can not be obtained, the gradient descent method is used to optimize the solution:

The first step: initialization of *W* and *B*; the second step: partial derivation of *W* and *B*:
  (7) $$\begin{array}{c} \frac{\partial E_{h}}{\partial W}=-B{\left(Y-W^{T}B\right)}^{T}+\lambda W, \end{array}$$
  (8) $$\begin{array}{c} \frac{\partial E_{h}}{\partial W}=-WY+\left(WW^{T}+\left(2+g\right)I\right)B-\left(\frac{2}{m}\right)BS. \end{array}$$
Step 3: update *W* and *B*
  (9) $$\begin{array}{c} W=W-\eta_{h1}\frac{\partial E_{h}}{\partial W}, \end{array}$$
  (10) $$\begin{array}{c} B=B-\eta_{h2}\frac{\partial E_{h}}{\partial B}. \end{array}$$
Step 4: bring the updated *W* and *B* into formula (6) and calculate the value. Continue iteratively performing step 1–step 4 until convergence.

#### 3.1.2. Experimental Setup

First, the most suitable *K* value is selected by experiment. As given in Table 4, the network achieves the best results when *K* = 5. Therefore, this experiment sets the number of network layers to 5.

Experimental performance measures are mean average accuracy (mAP). This performance metric is also widely used in other literature and is a general standard for retrieval tasks. Given a query sample, the average accuracy (AP) is calculated by

(11) $$\begin{array}{c} AP=\frac{1}{M}\sum_{\text{r}=1}^{R}\text{pre}\left(r\right)\text{rel}\left(r\right). \end{array}$$

#### 3.1.3. Results Analysis

As given in the table, the multimodal transport characteristics obtained by cascade direct method and the multimodal transport characteristics obtained by learning subsystem are poor in dimensional image search. The additional multimodal transport approach proposed in this section is more effective than other approaches. The combination of ciphers shows that the correlation and complementarity between different video data models with the best results in dimensional image search are very low. Results are given in Tables 5–7.

By nonlinearly transforming the properties of multimodule combinations into multimodal intermodal transport sensors, the method learns to express higher and more selective properties and automatically learns the relevance of each dimension to the semantics of outer space. The search work has increased due to the conversion of the learning method of characteristic changing hash monitoring into hash code, which keeps the similarity and difference within this category unchanged. Multimodal transport and hash learning methods are very effective.

### 3.2. Video Scene Classification Based on Consistency Semantic Learning

Different from the traditional video, the content of the video produced by different users is often very different, which is due to the subjectivity and randomness of the user image, resulting in the same image and the intention of expression.

#### 3.2.1. Method Description

Neural analytic network is an important method to learn various characteristics in computer vision. Excellent learning and performance in visual recognition of computer images and other visual missions ensure that the spatial characteristics of each image exit after passing the LSTM, the result of which is the concealment of each step; over time, it remains in the structure of the first layer of LSTM and is input as a LSTM unit at that time. The next procedure for calculating the hidden state can be found in the following formulas:

(12) $$\begin{array}{c} X_{ih}^{t}=O_{l}*\;\tanh\left(X_{ie}^{t}\right), \end{array}$$

(13) $$\begin{array}{c} x_{ic}^{\text{t}}=f_{t}*x_{ic}^{t-1}+i_{t}*{\tilde{x}}_{ic}^{t}\\ {\text{i}}_{\text{t}}=\sigma \left(W_{\text{xi}}x_{i}^{t}+W_{\text{hi}}x_{ih}^{t-1}+b_{\text{i}}\right)\\ {\text{o}}_{\text{t}}=\sigma \left(W_{\text{xo}}{\text{x}}_{i}^{t}+W_{\text{ho}}x_{ih}^{t-1}+b_{\text{o}}\right)\\ {\tilde{x}}_{ic}^{t}=\tanh\left(W_{xc}x_{i}^{t}+W_{hc}x_{ih}^{t-1}+b_{c}\right), \end{array}$$

where *α*_*t*_ is the weight of each frame without attention module that is automatically weighted by the inner product. The calculation process of weight and inner product is as follows:

(14) $$\begin{array}{c} \alpha_{t}=\frac{\exp\left(x_{ih}^{t}\right)}{\sum_{t=1}^{n}\exp\left(x_{ih}^{t}\right)}, \end{array}$$

(15) $$\begin{array}{c} Z_{\text{i}}^{t}=\langle \alpha_{\text{t}}{\text{,x}}_{\text{ih}}^{\text{t}}\rangle . \end{array}$$

After the attention module is weighted, the features of all frames pass through the LSTM layer again. LSTM layer is still a single-layer structure, and the output features of the layer are as follows:

(16) $$\begin{array}{c} z_{ih}^{n}=o_{n}*\;\tanh\left(z_{ic}^{n}\right), \end{array}$$

(17) zicn=f∗nzihn−1+in∗z˜icnon=σWzozin+Whozihn−1+boon=σWzozin+Whozihn−1+bofn=σWzfzin+Whfzihn−1+bn.

Each bit of the predicted category is shown in the following equation:

(18) $$\begin{array}{c} {\hat{\text{y}}}_{\text{i}}=\text{softmax}\left(W_{\text{fc}}{\text{z}}_{\text{ih}}^{\text{n}}+{\text{b}}_{\text{fc}}\right). \end{array}$$

To improve the consistency of microvideos in the same scenario, due to inconsistent content, this section adopts a two-sector framework and a supervised learning mechanism, while maintaining the similarity of scene categories between two-branch network parameters and samples. The forum where you want to post is a stage. The results L3 the stage cross-*L*1, *L*2 and relative loss functions are as follows:

(19) $$\begin{array}{c} L_{1}=-\sum_{i=1}^{Pl}\sum_{j=1}^{k}y_{ij}\ln\;{\hat{y}}_{ij1}+\left(1-y_{ij1}\right)\ln\left(1-{\hat{y}}_{ij1}\right), \end{array}$$

(20) $$\begin{array}{c} L_{2}=-\sum_{i=1}^{P\text{l}}\sum_{j=1}^{k}y_{ij2}\ln\;{\hat{y}}_{ij2}+\left(1-y_{ij2}\right)\ln\left(1-{\hat{y}}_{ij2}\right), \end{array}$$

(21) $$\begin{array}{c} L_{3}=\frac{1}{2Pl}\sum_{m=1}^{Pl}y_{m}d_{m}^{2}+\left(1-y_{m}\right)\max{\left(\text{m}\arg in-d_{m},0\right)}^{2}. \end{array}$$

The final objective function of the ACSL method is to minimize the weighted fusion of three losses. The calculation process of the objective function *L* is as follows:

(22) $$\begin{array}{c} \underset{W,B}{\min}L=\alpha L_{1}+\alpha L_{2}+\beta L_{3}, \end{array}$$

where *α* and *β* are the equilibrium parameters, and these two parameters are hyperparameters. *W* and *B* are the network learning parameters. The optimization method used in this section is the stochastic gradient descent method.

#### 3.2.2. Experimental Setup

In this section, the method network structure consists of two branches; each branch consists of two LSTM layers and one Attention layer, where the output dimension of the first LSTM layer is Q24, 1. The return_sequences “property value is True” indicates that all frames correspond to a 1024-dimensional output. And the output dimension of the second LSTM layer is 128, “The return_equences” property value is False, and it represents the final 128-dimensional output as given in Tables 8–10.

#### 3.2.3. Experimental Results

The comparison of performance of dual-branch and single-branch networks is given in Table 11. Validation of LSTM layers is given in Table 12.

This section introduces the classification of semantic learning Scopic models. This method utilizes the spatial characteristics of the video scene through a central pretraining image recognition network. Ensure the semantic expression ability of these features, combine the spatial characteristics with the time series characteristics adopted by the LSTM, and introduce the attention mechanism when extracting the time series characteristics. The semantic related content in the video box is automatically removed. Some table features are automatically weighted to obtain a more neutral table of spatial and temporal features. Through dual network and supervised learning mechanism, the consistency of video content in the same scene and the difference of time characteristics are maintained. Temporary experimental results confirm that this method is successful in classifying video.

### 3.3. Video Scene Classification Based on Multimodal Semantic Enhancement

Video, as a new form of media, is widely spread in social platforms. In addition to the visual information and sound information in the traditional video, the video has a good auxiliary effect on the scene understanding of the video. Therefore, the feature fusion of visual, sound, and text modes can learn rich scene feature representation.

Regarding the MESL method, the semantic enhancement of strong semantic modes to weak semantic modes is realized by minimizing the semantic distance between weak semantic modes and strong semantic modes and the discriminant loss of single modes. So, the semantic distance minimization objective function is shown in the following formula:

(23) $$\begin{array}{c} \underset{W,B}{\text{min}}\text{dis}=\alpha {\left\|y_{i}^{v\_\text{out}}{-}_{i}^{a\_\text{out}}\right\|}_{2}+\beta {\left\|y_{i}^{v\_\text{out}}-y_{i}^{t\_\text{out}}\right\|}_{2}. \end{array}$$

After the semantic enhancement, the cross-entropy loss of the three modes is calculated as follows:

(24) $$\begin{array}{c} \text{Loss}\_v=-\sum_{i=1}^{m}y_{i}^{v\_\text{out}}\ln\;y_{i}+\left(1-y_{i}^{v\_\text{out}}\right)\ln\left(1-y_{i}\right), \end{array}$$

(25) $$\begin{array}{c} \text{Loss}\_a=-\sum_{i=1}^{m}y_{i}^{a\_\text{out}}\ln\;y_{i}+\left(1-y_{i}^{a\_\text{out}}\right)\ln\left(1-y_{i}\right), \end{array}$$

(26) $$\begin{array}{c} \text{Loss}\_t=-\sum_{i=1}^{m}it\_\text{out}\ln\;y_{i}+\left(1-y_{i}^{t\_\text{out}}\right)\ln\left(1-y_{i}\right). \end{array}$$

Four trials are included in this section to validate the method of this section, that is, MESL, in the dimensional scenario classification. The first test was selected as a parameter to determine the equilibrium coefficient of the elements of the PE function. The second experiment is performance comparison to verify the comparison between the method and the method. The third trial is a combined trial to verify the effectiveness of weak semantic effectiveness and integration. The fourth test of multimodal transport is a confluence test to verify the consistency of the methods in this section and to evaluate the performance.

The integration method of multimodal transport is superior to that of single mode. The results of the combined tests are given in Tables 13 and 14. As given in Table 13, the characteristics of sound and text molds are superior to the previous version. The performance of the visual model decreases slightly, mainly because the semantic speech and text models are weak, but the visual model has not been improved, and the characteristics of multimodal transport are better than the single mode in the classification of microvideo images. Details are given in Tables 13 and 14.

In order to make full use of the semantic pattern contained in the weak semantic pattern, the method of this section adopts the method of strengthening the semantic pattern to deal with the weak semantic situation and enhances the expression ability of the semantic model characteristics. For microvideo teaching, in some cases, it is impossible to determine the scene type directly according to the way. However, the type of scene can be judged by sound or part of the text, which is very complementary to the lack of semantic model vision. Therefore, it is suggested that the enhanced multimodal transport microvideo images be classified by minimizing the semantic distance between the strong and weak modules and the interpretation errors of different molds. And we further integrate the characteristics before and after, in order to better integrate the strong model and the weak model semantically. Through the weight of self-study method and the automatic learning fusion between different weight modes, the experimental results suitable for the weight supplement of multimodal transport prove the effectiveness of the algorithm.

## 4. Requirements for Language Video Design

### 4.1. Video Should Focus on Students' Original Cognition

According to the students' learning foundation and ability, we must fully consider, study, and understand the confusion points and obstacles in practice, as well as the obstacles encountered in practice. Coverage should be as broad as possible, taking into account as many students as possible, for example, the use of video that links new learning materials to past knowledge, or new learning materials to knowledge acquired from student courses, and guides students to reflect in-depth dialogue and gradually improve their cognitive and ability. Whether in writing or onstage, or in determining the key and difficult points in the textbook, students must be able to understand and learn knowledge.

### 4.2. Video to Support Classroom Teaching Effectively

For students with strong motivation, video is very obvious. Teachers should be familiar with the teaching content of a subject or chapter and should be familiar with the overall planning and difficulties, problems, and requirements of teaching materials: accordingly, for example, regarding the teaching of ancient poetry, students may have difficulties in understanding words, customs, and the ancient system of rights. Before that, video can be systematically produced for students to use, covering classical Chinese, special phrases, vocabulary, and ancient words in textbooks. It can display the priorities, difficulties, and doubts of ancient poetry in the eyes of students, help them familiarize themselves with ancient philosophy and framework, understand the differences of ancient Chinese, and understand the rich connotation of Chinese culture.

### 4.3. The Practice of Chinese Subject Should be Emphasized by Video

Language learning is very practical, and teachers must guide students to deepen their understanding of PRA language learning methods. Science and technology teachers must combine teaching needs with students' learning conditions before preparing materials on video content, integrating practical content, and enriching and expanding video content. For example, once visual expression education is provided in the classroom, teachers can combine this education with writing education and carry out training activities that contribute to improvement of students' ability to write and write, create video design, download materials from the website, and connect different learning elements through various learning lines, in a way of presentation and narration, including a wide range of historical background, and landscape characteristics. On the one hand, it enables students to observe and understand in a visual and detailed way; on the other hand, it improves students' rationality and systematization. Video enables students to obtain vision, which has a positive impact on expanding students' thinking and enriching written materials.

## 5. Optimization of Teaching Strategy System Design of Video Language

### 5.1. Cognitive Theoretical Basis of Multimedia Learning

**Figure 1:**
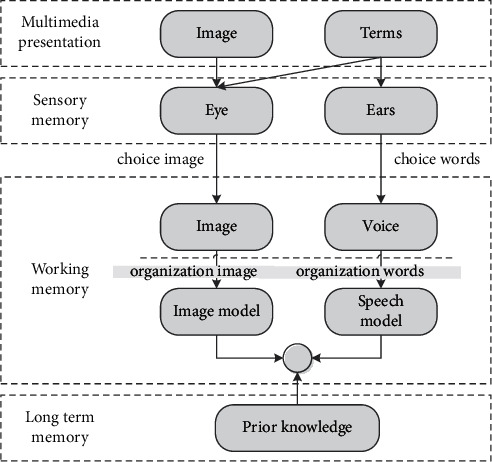
Cognitive model of multimedia learning.

Mayer cognitive theory of multimedia learning holds that meaningful learning must organize multimedia information according to different multimedia cognitive methods. The model shows that external information stimuli first enter sensory memory and then deliberately choose. From sensory memory, the results of multimedia cognitive experiments show that the use of multimedia has a positive impact on the retention and transfer of learning, which is superior to single learning. Multimedia display enables students to learn more deeply and understand learning content better, rather than putting learning content in a holistic form. Learning outcomes are better only when students focus enough working memory resources on useful information processing, as shown in Figure 1.

Through extensive practical research, the effectiveness of learning multimedia is proved. Video is a form of resources, combined with text and image, which is very important to image and sound and consistent with the meaning of multimedia learning. These characteristics are the basic concepts of this virtual view design research. Teaching videos spread knowledge through mixed music works and jointly display content. Educational video can be classified according to the prominence of sound and image and can also be used in the form of image, audio-assisted interpretation of the content, or in the form of illustrations. Mayer seven principles put forward media design, namely, multimedia knowledge, space proximity, time proximity, consistency, personal difference, and so on are helpful for understanding. They are the basis of current research on microinformation presentation design.

### 5.2. Elements of a Video for VARK Learning Styles

**Figure 2:**
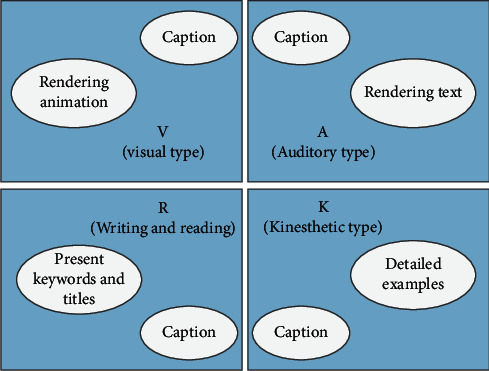
Classification of video elements for VARK learning style learners.

The video consists of two parts: image and sound: video learning (i.e., interaction of visual and auditory channels), information stimulation, and information processing, plus features of four types of Vark learning styles, in order to enable interaction between body organizations to better understand learning content. As shown in Figure 2, images and sounds reflect the uniqueness of each style. Video oriented to Vark learning styles should contain different contents, as shown in Figure 2.

### 5.3. Facilitating Information Processing for Learners

**Figure 3:**
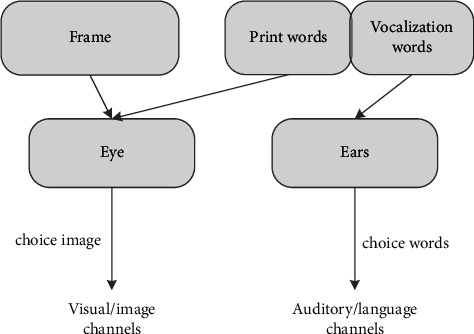
Information processing process model with redundant information.

Reducing the redundancy of information independent of learning content in video teaching design is helpful for students to process information and cognitive ability of redundancy effect according to multimedia learning theory. The principle of redundancy means that students can better learn visual materials composed of “animation” and “narration” rather than “animation,” “narration,” and “narration” of visual materials. “Screen text,” better knowledge in terms of migration, has too many animation effects, text, or subtitles and may be redundant, interfering with students' information processing: on the one hand, animation, text, and subtitle are received through the eyes, which results in visual overload of information; on the other hand, when the same information appears in visual and auditory form, the brain processing ability is limited, and processing two kinds of the same information reduces the processing of other useful information and increases cognitive ability. The internal Figure 3 shows an information processing model.

Based on the above findings, the study concluded that video should be designed to reduce excessive information and avoid interference and that these images should no longer appear in the same subtitle and recording or music background; on the basis of different characteristics of learning styles, visual video is presented in animation form, emphasizing visualization of abstract content, while the remaining acoustic, text, and kinetic view frequencies take the form of PPT video screens combined with demonstrations. PPT production designed many animation effects in order to be dynamic, but too many animation effects distracted the students' attention. As the basis of the principle of avoiding interference information redundancy, the complex influence of animation should be reduced as far as possible in design, only to gradually introduce some text and image characteristics, to reduce the simple influence of animation, without subtitles, and not to increase music.

### 5.4. Optimization of Teaching Strategy System Design for Microvideo Language

**Figure 4:**
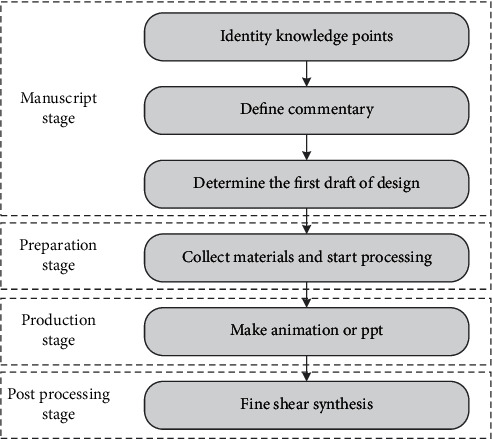
Production process for learning style microvideo.

**Figure 5:**
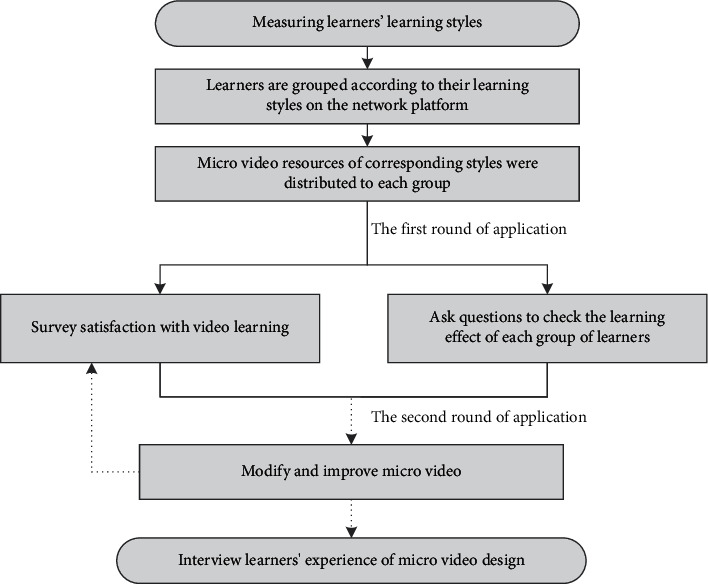
Microvideo resource application process diagram.

The microvideo development process for this study includes the drafting, preparation, fabrication, and subsequent processing phases, using the evaluation phase as a teaching practice. A project phase includes the identification of knowledge points and the definition of explanatory or explanatory text and the identification of design manuscripts; the preparatory phase includes the collection and processing of materials required for video production; the production phase of animation or PPT production materials; and the video synthesis phase, which simplifies video and audio production, processes the details of microvideo and improves the overall quality of microvideo.  Figure 4 shows the specific process. Details are shown in Figures 4 and 5.

Students will study through the network learning platform, before class to complete the microvideo learning, and in the after-class stage, teachers will randomly ask questions to check academic performance, to ensure the integrity of academic performance. Teachers randomly select one or two students from each learning style to ask questions and determine the satisfaction of all students with the use of microvideo. The results were tested by students' feedback to the class and the survey of school design. Use microvideo, and use satisfaction questionnaire and interview students when designing microvideo.

## 6. Conclusion

During the era of Internet + education, the emergence of mobile terminals such as smart phones and mobile blackboards provides strong teaching support for traditional language classes, supporting language teaching, with emphasis on changing the use of cumbersome and ineffective traditional teaching methods. Using interactive online learning platform to support students' active learning and cooperative learning, we can strengthen the ability of problem finding and questioning by implementing reasonable video teaching. Effective video teaching has injected the contemporary water of life into the development of language subject. If we further improve the quality of video teaching and production, provide students with a platform for interactive thinking and demonstration, and further enrich the means of dissemination of video, such as making video in language, in front-line language classes, language video will be more widely used.

## Figures and Tables

**Table 1:** Correlation between three modes.

Modality	Correlation coefficient
Visual and audio	0.5036
Visual and text	0.5069
Audio and text	0.1217

**Table 2:** Correlation between individual modes and scene categories.

Modality	Correlation coefficient
Visual	0.6135
Audio	0.1755
Text	0.2827

**Table 3:** Correlation between representation of three modes in common subspaces and categories.

Modality	Corrcoef_CCA	Corrcoef_MVDA
Visual	0.2502	0.2235
Audio	0.2074	0.0234
Text	0.2849	0.0964

**Table 4:** Comparison *K* network performance (mAP) at different values.

Values of *K*	mAP (@50)	mAP (@100)
*K* = 3	0.4255	0.4240
*K* = 4	0.4246	0.4200
*K* = 5	0.4504	0.4468
*K* = 6	0.4293	0.4298
*K* = 7	0.4253	0.4164

**Table 5:** Comparison of mAP performance of this section with traditional multimodal fusion methods.

Method	mAP (@50)	mAP (@100)
Concatenating	0.398	0.358
LDA	0.411	0.393
CCA	0.258	0.234
MvDA	0.282	0.250
Multilayer neural network	0.450	0.445
Proposed method	0.469	0.477

**Table 6:** Comparison of mAP@50 performance of methods in this section with individual hash learning methods.

Method	8 bits	16 bits	32 bits	64 bits
LFH	0.388	0.365	0.406	0.359
KSH	0.338	0.393	0.452	0.439
SDH	0.239	0.223	0.223	0.292
COSDISH	0.330	0.369	0.400	0.375
Proposed method	0.469	0.452	0.455	0.454

**Table 7:** Comparison of mAP@100 performance of this section method with individual hash learning methods.

Method	8 bits	16 bits	32 bits	64 bits
LFH	0.395	0.366	0.406	0.358
KSH	0.341	0.377	0.431	0.411
SDH	0.247	0.219	0.220	0.266
COSDISH	0.330	0.369	0.403	0.378
Proposed method	0.477	0.453	0.455	0.453

**Table 8:** Comparison of the performance of methods in this section with existing methods on Maryland datasets.

Class	HOF + GIST	SFA	C3D	ACSL
Avalanche	0.200	0.600	1.000	1.000
Boiling water	0.500	0.700	0.900	1.000
Chaotic traffic	0.300	0.800	0.900	1.000
Forest fire	0.500	0.100	0.800	1.000
Fountain	0.200	0.500	0.900	1.000
Iceberg collapse	0.200	0.600	1.000	0.800
Landslide	0.200	0.600	0.800	0.800
Smooth traffic	0.300	0.500	0.800	0.800
Tornado	0.400	0.700	0.800	0.800
Volcanic eruption	0.200	0.800	0.900	0.800
Waterfall	0.200	0.500	0.700	0.400
Waves	0.800	0.600	1.000	0.600
Whirlpool	0.300	0.800	0.900	1.000
Average	0.330	0.600	0.860	0.850

**Table 9:** Performance comparison of methods in this section with existing methods on Yupenn datasets.

Class	HOF + GIST	SFA	C3D	ACSL
Beach	0.870	0.930	0.970	1.000
Elevator	0.870	0.970	1.000	1.000
Fire	0.630	0.700	1.000	1.000
Fountain	0.430	0.570	0.830	1.000
Highway	0.470	0.930	0.970	0.890
Lightning	0.630	0.870	0.930	1.000
Ocean	0.970	1.000	1.000	1.000
Railway	0.830	0.930	0.970	1.000
Rfiver	0.770	0.870	1.000	0.890
Sky	0.870	0.930	0.970	1.000
Snowing	0.470	0.700	0.930	0.560
Street	0.770	0.970	1.000	0.890
Waterfall	0.470	0.730	0.970	0.890
Windmill	0.530	0.870	1.000	0.890
Average	0.680	0.850	0.970	0.930

**Table 10:** Performance comparison of methods in this section with existing methods on videoSceneData_10 datasets.

Class	HOF + GIST	SFA	C3D	ACSL
Museum	0.250	0.080	0.100	0.797
Pier	0.130	0.070	0.050	0.594
Garden	0.500	0.040	0.100	0.815
Office	0.030	0.020	0.050	0.594
Bridge	0.190	0.120	0.120	0.768
Racetrack	0.230	0.070	0.120	0.774
Landmark	0.210	0.060	0.050	0.788
Aquarium	0.300	0.470	0.050	0.818
Lake	0.060	0.070	0.120	0.683
Bowling alley	0.290	0.030	0.090	0.895
Average	0.220	0.100	0.100	0.753

**Table 11:** Comparison of performance of dual-branch and single-branch networks.

Class	Single-branch	Two-branch
Museum	0.774	0.797
Pier	0.622	0.594
Garden	0.877	0.815
Office	0.438	0.594
Bridge	0.752	0.768
Racetrack	0.744	0.774
Landmark	0.741	0.788
Aquarium	0.796	0.818
Lake	0.590	0.683
Bowling alley	0.860	0.895
Average	0.719	0.753

**Table 12:** Validation of LSTM layers.

Class	W/O-LSTM	W/LSTM
Museum	0.589	0.797
Pier	0.139	0.594
Garden	0.834	0.815
Office	0.775	0.594
Bridge	0.721	0.768
Racetrack	0.719	0.774
Laminuurk	0.668	0.788
Aquarium	0.776	0.818
Lake	0.500	0.683
Bowling alley	0.819	0.895
Average	0.684	0.753

**Table 13:** Modal semantic enhancement experiment.

Modal	Acc_before_enhancement	Acc_after_enhancement
Audio	0.3286	0.3427
Text	0.4153	0.4210
Visual	0.9816	0.9697

**Table 14:** Ablation experiment.

Modal	Accuracy
Audio	0.3427
Text	0.4210
Visual	0.9697
Visual + audio + text	0.9826

## Data Availability

The data used to support the findings of this study are available from the corresponding author upon request.
